# Altered Structural and Functional Connectivity of Juvenile Myoclonic Epilepsy: An fMRI Study

**DOI:** 10.1155/2018/7392187

**Published:** 2018-02-26

**Authors:** Chengqing Zhong, Rong Liu, Cheng Luo, Sisi Jiang, Li Dong, Rui Peng, Fuqiang Guo, Pu Wang

**Affiliations:** ^1^The Affiliated Hospital of Southwest Medical University, Luzhou, Sichuan 646000, China; ^2^Department of Neurology, The 452nd Hospital of PLA, Chengdu, Sichuan, China; ^3^Key Laboratory for NeuroInformation of Ministry of Education, School of Life Science and Technology, University of Electronic Science and Technology of China, Chengdu, Sichuan, China; ^4^Department of Neurology, Sichuan Provincial People's Hospital, 32 West Second Section First Ring Road, Chengdu, Sichuan 610072, China; ^5^Department of Neurology, Chongzhou People's Hospital, Chongzhou, Sichuan, China

## Abstract

The aim of this study was to investigate the structural and functional connectivity (FC) of juvenile myoclonic epilepsy (JME) using resting state functional magnetic resonance imaging (rs-fMRI). High-resolution T1-weighted magnetic resonance imaging (MRI) and rs-fMRI data were collected in 25 patients with JME and in 24 control subjects. A FC analysis was subsequently performed, with seeding at the regions that demonstrated between-group differences in gray matter volume (GMV). Then, the observed structural and FCs were associated with the clinical manifestations. The decreased GMV regions were found in the bilateral anterior cerebellum, the right orbital superior frontal gyrus, the left middle temporal gyrus, the left putamen, the right hippocampus, the bilateral caudate, and the right thalamus. The changed FCs were mainly observed in the motor-related areas and the cognitive-related areas. The significant findings of this study revealed an important role for the cerebellum in motor control and cognitive regulation in JME patients, which also have an effect on the activity of the occipital lobe. In addition, the changed FCs were related to the clinical features of JME patients. The current observations may contribute to the understanding of the pathogenesis of JME.

## 1. Introduction

Juvenile myoclonic epilepsy (JME) is characterized by transitory, bilateral symmetry, synchronous, and unrhythmic muscle jerks, generally, with no loss of consciousness [1]. The structural brain abnormalities are not found by conventional MRI and computed tomography in JME patients [1], but the development of hypersensitive neuroimaging technology illustrates the microstructural and functional changes of in JME patients [1]. Many alterations of subcortical structures have been reported in JME patients. Using proton magnetic resonance spectroscopy, the N-acetyl aspartate (NAA) levels of the thalamus were reduced in JME patients, which indicated thalamus dysfunction is part of epileptogenesis in JME [2]. In ^1^H-magnetic resonance spectroscopy (^1^H–MRS) studies, significantly reduced concentrations of NAA in the prefrontal were observed, which demonstrated the abnormality of the prefrontal cortex in JME patients compared with controls [3, 4]. When a VBM analysis was performed in JME patients, different results were observed in recent years. For instance, some studies found reduced GMVs in the prefrontal lobe [5, 6], the bilateral thalamus [7–11], supplementary motor area (SMA), the posterior cingulate gyrus [12], the insula, and bilateral cerebellar hemisphere [8]. Increased GMVs were found in the precentral gyrus, the parietal lobe, and the right middle temporal gyrus [13], and some studies even found increased GMVs in the frontal lobe. Of note, a recent study showed that the cerebellum had a decreased fractional anisotropy (FA) value in idiopathic generalized epilepsy, which demonstrated the microstructural integrity was changed [14]. At present, most of the VBM studies focused on the changes in the frontal lobe and thalamus; however, an increasing number of abnormal structural changes have been found in other brain regions.

To further understand the pathogenesis of JME, the resting state FC has proven to be an effective means for investigating the characteristics of brain activity, and it has been widely used to study epilepsy in recent years [15–17]. Vulliemoz et al. and Vollmar et al. demonstrated increased FC between the motor cortex and SMA based on cognitive task analysis in JME patients [18, 19], which hinted that cognitive activity triggered the myoclonic jerks [20]. Electrophysiological and functional neuroimaging studies suggested a functional disorder in the fronto-thalamo-frontal circuitry [2, 21] in JME patients. Synchronous electroencephalography and functional magnetic resonance imaging (EEG-fMRI) studies have demonstrated the generalized spike and wave discharge-related thalamic and default mode network (DMN) were deactivated in idiopathic generalized epilepsy (IGE); meanwhile, clear activation in the cerebellum has also been recorded during epileptic discharges [22]. Moreover, a reduced regional homogeneity (ReHo) value in the cerebellum was found in our previous study [23] using the rs-fMRI analysis in JME patients, which suggested the underlying spontaneous activity of the cerebellum is partially related to the pathogenesis of JME. Previous studies considered that the interactions between cortical and subcortical structures are important for the generation of generalized epilepsy [24, 25]. Therefore, epilepsy is regarded as a disruption of functional networks [26].

Previous studies have found the thalamo-frontal network plays an important role in the pathogenesis of JME. Although there have been many studies on the pathogenesis of JME, it is not fully elucidated. We therefore combined VBM and FC analysis using high-resolution T1 and rs-fMRI data to provide more evidence for understanding the potential pathophysiological mechanisms of JME.

## 2. Methods

### 2.1. Participants

Twenty-five patients (mean age: 24.68 ± 6.4 years, mean years of duration: 12.16 ± 7.4, and mean age of seizure onset: 12.16 ± 3.9 years, 17 females) with JME were recruited in the Center for Information in Medicine, University of Electronic Science and Technology of China. The diagnosis of JME was in line with clinical histories and electroencephalographic findings and was consistent with the International League Against Epilepsy (ILAE) classifications [1] (C. Zhong, P. Wang, F. Guo). All the conventional neuroimaging examinations, including CT and MRI scanning, displayed no structural abnormalities by visual inspection. Twenty-four healthy subjects were recruited as the sex- and age-matched control group (mean age: 25.41 ± 7.7 years; 15 females). None of the controls had neurological or psychiatric disorders. This study was approved by the ethical committee of the University of Science and Technology of China according to the standards of the Declaration of Helsinki. Every patient provided signed informed consent.

### 2.2. Data Acquisition

All brain MR data was collected by a 3T GE MRI scanner with an eight-channel-phased array head coil (MR750; GE Discovery, Milwaukee, WI) at the MRI research center of the University of Electronic Science and Technology of China. The T1-weighted images were acquired using a three-dimensional fast spoiled gradient echo (T1-3DFSPGR) sequence (TR = 6.008 msec, TE = 1.984 msec, FA = 90°, matrix = 256 × 256, FOV = 25.6 × 25.6 cm^2^, and slice thickness (no gap) = 1 mm). Rs-fMRI data were acquired using gradient echo EPI sequences (TR = 2000 ms, TE = 30 ms, FA = 90°, matrix = 64 × 64, FOV = 24 × 24 cm^2^, slice thickness = 4 mm (no gap), and 35 slices per volume). All subjects underwent a 510 s resting state scan yielding 255 volumes. Subjects were asked to close their eyes and not sleep during the data scanning [27].

### 2.3. Voxel-Based Morphometry Analysis

The analysis of voxel-based morphometry was performed using the SPM8 package, which was implemented in MATLAB R2014 (available from http://dbm.neruo.uni-jena.de/vbm/). First, the obtained DICOM data was transferred into a nifit file, then the artifacts in all pictures were checked, and the images were repositioned to verify the origin of coordinates on the joint of anterior commissure.

Second, a diffeomorphic nonlinear registration algorithm (DARTEL, diffeomorphic anatomical registration through exponentiated lie algebra) was used to deform the GM partitions into a new customized reference space representing an average of all the subjects [28]. All T1 images were manually coregistered with the standard T1 template provided by SPM8. The coregistered images were then divided into GM, white matter (WM), and cerebrospinal fluid (CSF) using the unified segmentation procedure [29]. The resulting average size images were ulterior spatially normalized to Montreal Neurological Institute (MNI) space.

Third, the volume deformation caused by the nonlinear change was corrected with the Jacobian determents, and an isotropic Gaussian kernel (8 mm full width at half maximum) was used to smooth the modulated GM images.

Finally, the GMVs of the JME patient were compared with the normal control group using two sample *t*-tests in SPM8. The age and gender were corrected as confounding covariates. The level of significance for group differences was set at *p* < 0.05 (FDR corrected). The identification of regions of interest (ROI) was based on the comparison of GMV.

### 2.4. Functional Connectivity Analysis

RS-fMRI data were preprocessed using the SPM8 software package (http://dbm.neruo.uni-jena.de/vbm/). The functional data were corrected for slice timing and head motion. To avoid the influence of magnetic field instability, the first five volumes were removed. Subjects with head motion more than 3 mm (translation) and 1.5° (rotation) during scanning were excluded. Next, the corrected imaging data were spatially normalized to standard MNI space and resampled to 3 × 3 × 3 mm^3^. Then, spatial smoothing was applied with an 8 mm full-width at half maximum (FWHM) Gaussian kernel and bandpass filter (0.01–0.08 Hz) that dealt with the timing sequences. Finally, head motion parameters, CSF and WM, and globe signals were regressed out in the general linear model for each subject. After preprocessing of rs-fMRI data, the seeds were defined with a radius of 3 mm and a center voxel at the peak of the between-group difference in VBM analysis. For a given seed, the functional connectivity was calculated between the time courses from each seed and voxel in the whole brain using REST software (http://www.restfmri.net). For each group, an individual functional connectivity map was analyzed with a random effect one-sample *t*-test to identify voxels that showed a significant correlation with the seed. The two-sample *t*-test was used to compare the functional connections between groups. The group comparison was restricted to an explicit mask from the union set of the significant one-sample *t*-test results. Furthermore, a correlation analysis was performed to detect the relation between the FC that showed a significant between-group difference and the clinical features, including age of seizure onset and duration of epilepsy, with controlling effects of gender and age [27].

## 3. Results

### 3.1. Demographic and Clinical Characteristics

There were no significant differences in age or sex between the patients and controls. The detailed demographic information of patients is shown in Table 1. No one was excluded because of excessive head motion.

### 3.2. Voxel-Based Morphometric Analysis

**Figure 1:**
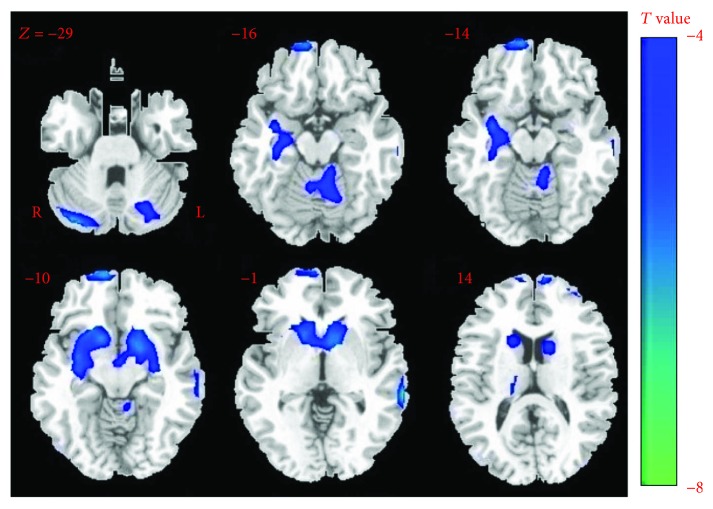
The difference of GM volume between groups. The decreased volume in patients contrasting to healthy controls is shown in cool color. No GM volume increase was observed in patients.

Compared with the control group, patients with JME showed significantly decreased GM volumes of regions in the cerebellum (bilateral anterior of cerebellum and vermis), the right thalamus, bilateral caudate, part of the cerebral cortex (the orbital superior frontal gyrus and left middle temporal gyrus), the right hippocampus, and the left putamen (Table 2, *p* < 0.05, FDR corrected) (Figure 1). We used the xjView toolbox (http://www.alivelearn.net/xjview) to visualize the results. No brain regions showed increased gray matter volume in patients with JME. Thus, based on the results from VBM, we chose ten ROIs for the following functional connectivity analysis.

### 3.3. Functional Connectivity Analysis

**Figure 2:**
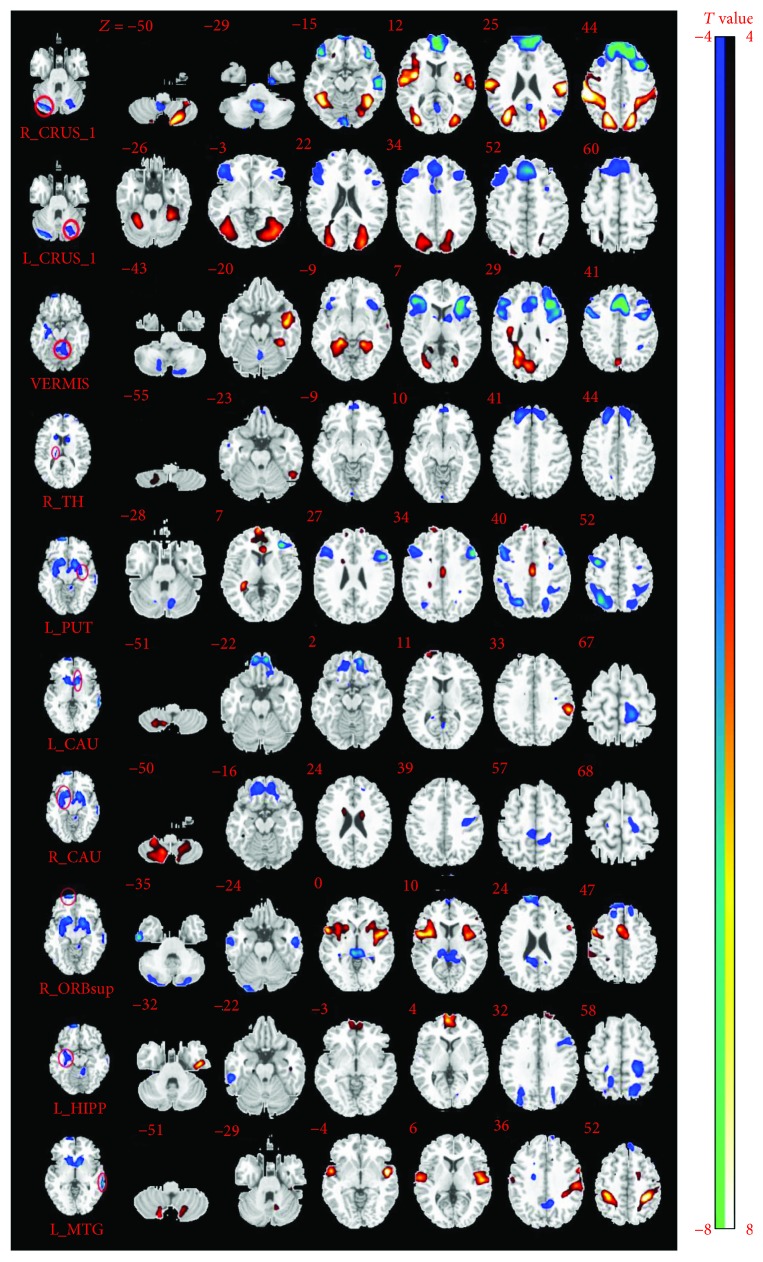
The different functional connection (FC) between groups. The position of seeds in AAL regions is shown on the left side of the figure. Each row shows changed FCs with left seed. The hot color means increased FCs, and cool color represents decreased FCS with the seeds.

Resting state FC analysis revealed the altered functional connections with selected ROIs (*p* < 0.05, FDR corrected; Figure 2). For the seed at the right anterior cerebellum (MNI coordinate: *x* = 34, *y* = −84, and *z* = −31), patients showed significantly increased FCs with the left posterior cerebellum, right middle occipital gyrus, left superior occipital gyrus, and bilateral postcentral gyrus, and decreased FCs with the left superior frontal gyrus, bilateral orbital inferior frontal gyrus, left middle temporal gyrus, and the left SMA. For the left anterior cerebellum (MNI coordinate: *x* = −26, *y* = −71, and *z* = −29), increased FCs were found with the cluster of the left middle occipital gyrus, the cluster of the right superior occipital gyrus, and the bilateral anterior cerebellum. Decreased FCs to the left anterior cerebellum were also found in the cluster of the right middle frontal gyrus, the right SMA, the left orbital inferior frontal gyrus, and the left middle frontal lobe. For the seed at the vermis (MNI coordinate: *x* = 6, *y* = −55, and *z* = −22), patients showed increased functional connections with the left middle temporal gyrus, the left fusiform gyrus, and the cluster of the right precuneus lobule and decreased FCs with the left SMA, the clusters of the bilateral triangular inferior frontal gyrus.

For the left putamen (MNI coordinate: *x* = −18, *y* = 1, *z* = −10), patients showed increased FCs with the right hippocampus and the left middle cingulate gyrus and decreased FCs with the bilateral triangular inferior frontal gyrus, the right precentral gyrus, the bilateral superior parietal lobule, and the left inferior parietal lobe. For the left caudate (MNI coordinate: *x* = −10, *y* = 12, and *z* = −5), patients showed increased FCs with the right inferior cerebellum and the left supramarginal gyrus and decreased FCs with the left orbital superior frontal lobe and the left paracentral lobule. For the right caudate (MNI coordinate: *x* = 12, *y* = 21, and *z* = 3), patients showed increased functional connections with the bilateral posterior cerebellum and the bilateral caudate. Decreased functional connections to the right caudate were also found in the right orbital superior frontal gyrus and the left postcentral gyrus. For the seed at the right thalamus (MNI coordinate: *x* = 18, *y* = −23, and *z* = 13), patients showed increased functional connections with the right posterior cerebellum and the bilateral thalamus and decreased FCs with the right superior frontal gyrus and left medial superior frontal gyrus.

For the seed at the right orbital superior frontal gyrus (MNI coordinate: *x* = 10, *y* = 64, and *z* = −13), patients showed increased FCs with the bilateral insula gyrus and the bilateral middle cingulate gyrus and decreased FCs to the right anterior cerebellum, the right inferior temporal gyrus, the left middle temporal gyrus, vermis, and the right medial superior frontal gyrus.

For the seed at the left middle temporal gyrus (MNI coordinate: *x* = −67, *y* = −33, and *z* = −44), patients showed increased FCs with the clusters of the bilateral superior temporal gyrus, clusters of the left inferior parietal lobule, and clusters of the right postcentral gyrus. FCs were decreased to the left middle temporal gyrus with the right anterior cerebellum, the left precuneus lobule, and the left medial superior frontal lobe. For the right hippocampus (MNI coordinate: *x* = 30, *y* = −8, and *z* = 16), patients showed increased FCs with the left inferior temporal gyrus and clusters of the bilateral medial superior frontal gyrus and decreased FCs with the right inferior temporal gyrus, the left postcentral gyrus, the right Rolandic operculum, and the bilateral occipito-parietal lobules. These differences in functional connections are fully visualized in Table 3.

### 3.4. The Correlation Analysis between Functional Connections and Clinical Features, Including Onset Age and Duration of Epilepsy

The FC in the brain regions with significant between-group differences were partially related to the duration and onset age in JME patients, while controlling for age, gender, and head motions. First, there were no significant relationships between the VBM and either the duration or age of onset in JME patients.

**Figure 3:**
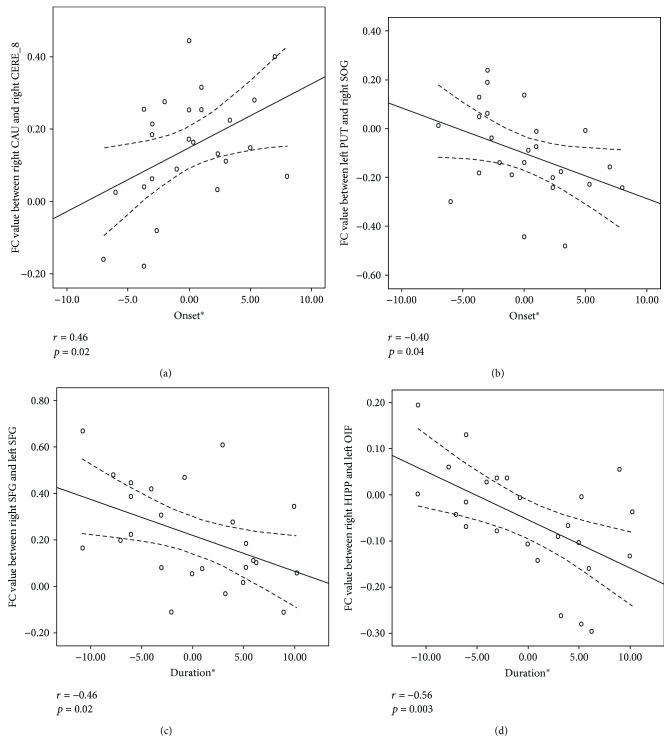
The correlation between FCs and clinical features. The FC between the right caudate and right cerebellum crus 8 showed positive correlations with the onset of age in JME patients (*r* = 0.46, *p* = 0.02). (a) FC between left putamen and right occipital superior gyrus showed negative correlations with the onset of age (*r* = −0.04, *p* = 0.04). (b) FCs between left superior frontal gyrus and right superior frontal gyrus showed negative correlations with the illness duration (*r* = −0.46, *p* = 0.02). (c) And FCs between right hippocampus and right orbital inferior frontal gyrus (*r* = −0.56, *p* = 0.003) (d) also showed negative correlations with the illness duration of JME.

For functional connectivity, significant positive correlations with onset age were found in the FCs between the right caudate and the right posterior cerebellum (MNI coordinate: *x* = −14, *y* = −68, and *z* = −45) (*r* = 0.46, *P* = 0.02, Figure 3(a)), but there was a negative correlation in FCs between the left putamen and the right occipital superior gyrus (*r* = −0.04, *P* = 0.04, Figure 3(b)). In addition, FCs between the left superior frontal gyrus and the right superior frontal gyrus (*r* = −0.46, *P* = 0.02, Figure 3(c)) and the FCs between the right hippocampus and the right orbital inferior frontal gyrus (*r* = −0.56, *P* = 0.003, Figure 3(d)) showed negative correlations with the illness duration of JME.

## 4. Discussion

In the present study, we investigated alterations of the brain structure and functional connections in JME patients. Compared with the control group, the main findings in JME patients were as follows: (1) The decreased GMVs were mainly located at the cerebellum, the basal ganglia, the thalamus, and parts of the cerebral cortex including the frontal area, the parieto-occipital lobe, the left temporal lobe, and the memory-related hippocampal gyrus. (2) In the functional connectivity analysis, we found significantly increased FCs in the motor-related and sensory-related areas, decreased FCs in the cognitive-related areas, and changed FCs in the language- and memory-related regions. (3) We also found significant differences in FCs in the brain regions that were correlated with onset age and duration of epilepsy in JME patients.

### 4.1. Alterations in Motor-Related Regions

Previous studies suggested that the anterior cerebellum participated in feedforward motor control, and the posterior cerebellum participated in transforming visual information feedback into precise motor adjustments [30]. Researchers have demonstrated that the cerebellum receives a large amount of information widely from the cerebral cortex [31, 32], then its efferents, with GABAergic inhibition via the thalamus, project to the primary motor cortex, and the premotor cortex participates in movement regulation [33], while the efferents project to prefrontal and posterior parietal areas participate in cognition and visuospatial function [34, 35]. In epilepsy, some studies have demonstrated structural and functional alterations in the cerebellum. For example, reduced fractional anisotropy values were detected in the right cerebellum, and reduced FCs between the right cerebellar and the left middle frontal gyrus were also detected [14, 36]. In the current study, a decreased GMV of the bilateral anterior cerebellum, increased FCs between the posterior cerebellum and the right thalamus, and even increased FCs between the anterior cerebellum and the posterior cerebellum were found, which might be related to the function of feedforward motor control [37]. These findings may indicate that the increased activity of the cerebellum, accompanied by the enhanced abnormal movement in JME patients, reflected the feedback mechanism in movement regulation. In addition, the findings showed disruption of the cerebello-thalamo-cortical circuits in JME patients. We also observed decreased FCs between the cerebellum and SMA. The SMA usually suggests involvement in the postural stabilization of the body, coordination, and movement [38, 39]. Thus, we presumed that the decreased FCs may testify declined activation of the coordination area.

For the motor-related basal ganglia regions, the left putamen and the bilateral caudate showed decreased GMV and increased FCs between the bilateral caudate and the posterior cerebellum. The basal ganglia are widely considered to play an important role in the regulation of discharges [40]. The caudate is especially involved in interictal spike-wave discharges and generalized seizure activity, which was found by Li et al. [41]. In addition, our findings of increased FCs between the caudate and the posterior cerebellum revealed a modulation effect on the activity of epilepsy, because of the feedback effect of the posterior cerebellum in motor regulation mechanisms. These findings are in line with our previous study of the basal ganglia network in idiopathic generalized epilepsy (2012) [42]. In addition, we observed the FCs between the right caudate and the right posterior cerebellum were positively related to the onset age in JME patients.

Furthermore, significantly increased FCs between the anterior cerebellum and the occipital lobe were observed in the JME patients. The occipital lobe mainly manifested as photosensitive property in epilepsy patients, especially in idiopathic occipital lobe epilepsies [43]. An existing study showed that 30% of JME patients are photosensitive [44]. However, there were no definite photosensitive seizures in all patients recruited in the current study. This phenomenon may be understood by the increased FCs with the cerebellum, which affected the function of the occipital cortex and inhibited the function of the cerebellum. The FCs between the left putamen and the right occipital superior gyrus were negatively related to the onset age in JME patients, and this may further hint to photosensitivity seizure reduction following the increased FCs with the cerebellum.

### 4.2. Alterations in Cognition-Related Regions

The efferents of the cerebellum project to prefrontal areas and posterior parietal areas that participate in cognition and visuospatial functions [45, 46]. There is hard evidence that has suggested the function of the frontal lobe mainly manifests as working memory [47], executive functions [48], and prospective memory [49]. In our study, the reduced GMV of the right orbital superior frontal gyrus and decreased FCs between anterior cerebellum and frontal lobe in JME patients might imply the cognitive activity declination. This phenomenon may be caused by the preponderant discharges of the frontal lobe [50] in JME, which lead to the decreased GMV of local neuron or neuron cell impairment [51, 52]. Meanwhile, the decreased FCs between the bilateral superior frontal gyrus in patients is negatively related with the illness duration (Figure 3(c)). This finding may demonstrate that the cognitive activity gradually aggravated by the extension of disease duration. Besides, we also found the right anterior cerebellum and the bilateral intraparietal sulcus (IPS) showed significantly increased FCs (showed in Figure 2), and the IPS is known as important nodes of the dorsal attention network (DAN) [53], which influence the visual area when attended to spatial orienting [54]. In addition, the increased FCs for anterior cerebellum and vermis to the precuneus has been founded, and the latter also involves in visuospatial processing [55]; these findings may further suggest the increased activity of cerebellum in the regulation of visuospatial plan in JME patients.

Another remarkable finding in our study is the decreased GMV of the right superior orbital frontal gyrus and increased FC between the right superior orbital frontal gyrus and bilateral insula. The insula is usually involved in consciousness, emotion, and the body's homeostasis, which are the nodes of the salience network (SN) and play a crucial role in switching between the default mode network and central executive network [56]. This is consistent with the previous diffusion tensor imaging study, which revealed the insula interconnected to orbitofrontal cortex [57]. The increased FCs represent the increasing activity of insula and superior orbital frontal gyrus, which would inflect the enhanced interaction within limbic system in JME. Alternatively, it may owe to the more anxiety/arousal during MRI scanning [58] in patients than healthy controls.

Taken together, these findings suggest that the regulation ability of cerebellum in cognition was decreased in JME patients, and decreased FCs between cognitive-related regions in JME patients were also found.

### 4.3. Alterations in Hippocampus and Temporal Lobule

Temporal lobe, especially mesial temporal structure, was affected by epilepsy activity in different types of epilepsy. In this study, the decreased GMV of the right hippocampus was observed. Consistent with our findings, the atrophy of hippocampus has already been described in JME patients in the previous study [5]. The potential reason included that the production of new neurons can be negatively affected by epileptic seizures [59], and the hyperexcitability can result in cytotoxicity and cell death [51].

Furthermore, we found decreased GMV in the left middle temporal gyrus which shows increased FC with left inferior parietal lobule, right postcentral gyrus, and left superior temporal gyrus. Evidence showed the left temporal lobe takes part in auditory and visual processing streams [60]. The postcentral gyrus is located at the primary somatosensory cortex, which is concerned with the main sense of touch. The function of these areas mainly involves in language, auditory processing, and sense of touch. Thus, we presume that the increased FC between these regions may reflect the compensation mechanism between functional similarity regions [61].

Notably, there are some shortcomings in our study. Firstly, the subjects are relatively small, so we cannot group it and discuss it further. If a group of patients with untreated epilepsy was involved in the control, there should be a more comprehensive explanation for the results. The sample size should gradually increase as our research continues. Secondly, if combined with EEG detect in the fMRI scanning, it would be better to access the correlation between epileptiform discharge and cognitive. In addition, the antiepileptic drugs may have some effect on the discharge of neuron and cognition; meanwhile, we also believe that comparing plus-treatment patients with epilepsy directly to a group of healthy patients can reflect the effects of epilepsy itself on the brain [62]. We would attempt to reduce these defects as far as possible in the future and improve the method. Finally, intelligence and mental test should be accomplished in the future work.

## 5. Conclusion

In summary, patients with JME showed focal structure abnormalities, especially the cerebellum, and found changed FCs in the motor-related area and cognitive-related regions. These findings revealed the important role of cerebellum in the pathogenesis of JME and may help us deeply understand the dysfunction in JME patients.

## Figures and Tables

**Table 1:** Demographic data of 25 juvenile myoclonic epilepsy patients.

Number	Gender	Age (years)	Age at seizure onset (year)	Antiepileptic drugs	History/family history
1	F	17	13	VPA	Sister with JME
2	F	17	13	VPA	Sister with JME
3	M	26	8	MVP	None
4	M	21	17	LEV	Grandmother with epilepsy
5	F	20	6	VPA, LTG	None
6	F	34	14	—	None
7	F	27	16	—	Daughter with epilepsy
8	F	16	13	LTG	None
9	M	30	9	CBZ, LEV	None
10	M	34	14	VPA, VPM	None
11	F	25	10	VPA	None
12	F	22	14	VPA	None
13	F	24	18	CBZ, VPA	None
14	M	37	12	VPA	None
15	F	23	7	VPA	None
16	F	17	10	VPA	None
17	F	29	10	VPA, VPM	None
18	F	33	20	VPM, LTG	None
19	M	18	14	VPM	None
20	F	25	21	VPM	None
21	M	22	15	VPA	None
22	M	38	8	VPA	Brother with epilepsy
23	M	22	8	VPM	None
24	F	21	11	VPM	None
25	F	19	12	MVP	Uncle with IGE

M: male; F: female; VPA: valproic acid; VPM: valpromide; MVP: magnesium valproate; LTG: lamotrigine; TOP: topiramate; CBZ: carbamazepine.

**Table 2:** The cluster with decreased GM volumes in patients with JME compared with controls.

Regions	Abbreviation	L/R	MNI coordinates			Peak *T* value	Cluster size
			*x*	*y*	*z*		
Cerebellum_crus1	CERE_CRUS 1	R	34	−84	−31	−8.20	79
Cerebellum_crus1	CERE_CRUS 1	L	−26	−71	−29	−4.46	—
Frontal_sup_Orb	ORBsup	R	10	64	−13	−6.50	82
Temporal_Mid	MTG	L	−67	−33	−4	−7.57	78
Putamen	PUT	L	−18	1	−10	−6.03	—
Hippocampus	HIPP	R	30	−8	16	−4.80	—
Caudate	CAU	L	−10	12	−5	−5.84	354
Caudate	CAU	R	12	21	3	−5.65	—
Vemis_4_5	CBV	R	6	−55	−22	−5.00	124
Thalami	TH	R	18	−23	13	−5.03	28

L: left; R: right.

**Table 3:** Altered intrinsic functional connectivity seeded at regions with decreased GM volume in patients with JME compared with controls. CRUS 1: cerebellum crus 1; ORBsup: superior orbital frontal gyrus; MTG: middle temporal gyrus; PUT: putamen; HIPP: hippocampus; CAU: caudate; CBV: cerebellum vermis; TH: thalamus. L: left; R: right.

ROI	Brain region	MNI coordinates			*T* value	Voxel number
		*x*	*y*	*z*		
R_CRUS_1	Occipital_Mid_R	27	−65	27	6.33	1027
	Occipital_Sup_L	−23	−69	36	4.84	777
	Cerebellum_8_L	−25	−69	−52	4.87	120
	Postcentral_R	58	−15	26	4.79	
	Parietal_Sup_R	18	−70	47	5.46	
	Postcentral_L	−33	−36	47	4.36	
	Frontal_Sup_L	−19	21	54	−3.68	1054
	Temporal_Mid_L	−56	−29	−9	−4.94	271
	Frontal_Inf_Orb_L	−41	34	−18	−4.72	130
	Frontal_Inf_Orb_R	42	36	−18	−5.26	72
	Supp_Motor_Area_L	−8	21	64	−6.44	
L_CRUS_1	Occipital_Mid_L	−27	−85	22	5.84	699
	Occipital_Sup_R	28	−83	22	5.44	497
	Cerebellum_6_R	28	−53	−26	5.93	
	Cerebellum_6_L	−32	−44	−26	6.29	
	Frontal_Mid_R	46	26	39	−4.45	744
	Frontal_Mid_L	−36	28	39	−4.14	175
	Frontal_Inf_Orb_L	−48	36	−7	−3.81	114
	Supp_Motor_Area_R	7	22	61	−4.38	
CBV	Temporal_Mid_L	−48	2	−21	3.67	96
	Fusiform_L	−36	−45	−18	4.85	148
	Precuneus_R	18	−52	24	4.83	232
	Supp_Motor_Area_L	−5	18	45	−6.46	525
	Frontal_Inf_Tri_L	−36	30	0	−6.18	411
	Frontal_Inf_Tri_R	48	33	6	−4.88	336
R_TH	Cerebellum_8_R	18	−51	−60	3.92	50
	Thalamus_R	18	−15	12	3.81	25
	Thalamus_L	−15	−15	9	3.68	28
	Frontal_Sup_R	15	39	39	−5.63	174
	Frontal_Sup_Medial_L	−10	61	16	−3.93	149
R_CAU	Cerebellum_8_R	12	−60	−54	5.95	346
	Cerebellum_8_L	−14	−68	−45	4.50	171
	Caudate_L	−15	−3	21	4.39	35
	Caudate_R	15	3	21	4.23	30
	Frontal_Sup_Orb_R	12	52	−22	−5.04	168
	Postcentral_L	−40	−16	36	−4.22	100
L_CAU	Cerebellum_8_R	12	−66	−51	3.69	54
	SupraMarginal_L	−57	−33	30	4.77	107
	Paracentral_Lobule_L	−13	−26	78	−4.50	74
	Frontal_Sup_Orb_L	−13	44	−24	−3.48	68
L_PUT	Cingulum_Mid_L	−3	−15	36	4.38	55
	Hippocampus_R	36	−30	−6	4.80	32
	Frontal_Inf_Tri_L	−36	39	3	−5.78	65
	Precentral_R	36	1	51	−5.17	83
	Frontal_Inf_Tri_R	−53	19	29	−5.21	86
	Parietal_Sup_L	−24	−68	43	−3.46	122
	Parietal_Inf_L	−30	−46	45	−3.09	111
	Parietal_Sup_R	28	−64	53	−4.02	187
R_ORBsup	Insular_L	−40	0	−5	4.59	234
	Insular_R	54	5	6	5.09	284
	Cingulum_Mid_R	10	11	39	4.57	124
	Cerebellum_Crus1_R	32	−86	−30	−3.88	44
	Temporal_Inf_R	57	−10	−33	−4.57	80
	Temporal_Mid_L	−52	−9	−24	−3.78	55
	Vermis_3	0	−35	−3	−3.86	124
	Frontal_Sup_Medial_R	9	62	30	−4.34	160
L_MTG	Temporal_Sup_R	54	0	−6	4.38	190
	Temporal_Sup_L	−51	−2	−6	6.13	246
	Parietal_Inf_L	−34	−39	45	5.99	222
	Postcentral_R	36	−42	63	4.79	151
	Cerebellum_Crus1_R	13	−79	−25	−4.71	215
	Precuneus_L	−9	−54	33	−3.77	74
	Frontal_Sup_Medial_L	−9	39	45	−3.85	36
R_HIPP	Temporal_Inf_L	−47	−16	−34	4.66	61
	Frontal_Sup_Medial_R	4	61	6	4.88	127
	Frontal_Sup_Medial_L	−1	60	6	4.54	
	Temporal_Inf_R	57	−33	−27	−4.16	42
	Rolandic_Oper_R	64	−4	12	−4.00	32
	Parietal_Sup_L	−21	−66	47	−4.11	145
	Occipital_Mid_R	32	−79	30	−3.69	65
	Postcentral_L	−22	−35	63	−3.82	140
	Parietal_Sup_R	16	−50	59	−3.65	47
